# Growth of *Mycobacterium avium* subsp. *paratuberculosis*, *Escherichia coli*, and *Salmonella* Enteritidis during Preparation and Storage of Yogurt

**DOI:** 10.1155/2013/247018

**Published:** 2013-12-16

**Authors:** K. Cirone, Y. Huberman, C. Morsella, L. Méndez, M. Jorge, F. Paolicchi

**Affiliations:** ^1^Laboratorio de Bacteriología, Grupo Sanidad Animal, EEA INTA, 7620 Balcarce, Argentina; ^2^Facultad Ciencias Agrarias, UNMdP, CC 276, 7620 Balcarce, Argentina; ^3^Facultad Ciencias Veterinarias, UNCPBA, Campus Universitario, Paraje Arroyo Seco, 7000 Tandil, Argentina

## Abstract

The purpose of this study was to determine the viability of *Mycobacterium avium* subsp. *paratuberculosis* (MAP), *Escherichia coli* (*E. coli*), and *Salmonella* Enteritidis (*S.* Enteritidis) during preparation and refrigerated storage of yogurt. Three yogurts were prepared using pasteurized commercial milk. Each yogurt was artificially contaminated with (1) MAP, (2) *E. coli* + *S.* Enteritidis, and (3) MAP + *E. coli* + *S.* Enteritidis. Samples were taken during and after the fermentation process until day 20 after inoculation. MAP was not detected during their preparation and short-term storage but was recuperated after starting at 180 min after inoculation storage. Live bacterial counts of *E. coli*, and *S.* Enteritidis increased during the first 24 hours, followed by a slight decrease towards the end of the study. In this study it was shown how MAP, *E. coli*, and *S.* Enteritidis resisted the acidic conditions generated during the preparation of yogurt and low storage temperatures. This work contributes to current knowledge regarding survival of MAP, *E. coli*, and *S.* Enteritidis during preparation and refrigerated storage of yogurt and emphasizes the need to improve hygiene measures to ensure the absence of these pathogenic microorganisms in dairy products.

## 1. Introduction

*Mycobacterium avium *subsp.* paratuberculosis* (MAP) is the causative agent of paratuberculosis or Johne's disease. MAP affects domestic and wild animals and, in cows, causes chronic enteritis, diarrhea, weight loss, and progressive emaciation that can eventually lead to death [1]. MAP has also been linked to human Crohn's disease, a systemic disorder that causes mainly a chronic inflammation of the intestine [2]. It is suggested that humans might be infected through contaminated milk, although relatively little is known about MAP survival during industrial milk manipulation. Some authors have suggested that pasteurization is capable of destroying mycobacteria. Thus, laboratory assays were performed to evaluate MAP heat resistance according to differential distribution of heat treatment during pasteurization [3–6]. In contrast, other authors support the theory that MAP is able to resist pasteurization when it is present in raw milk [7–13].

Viable MAP was detected in commercial pasteurized milk in the UK, the USA, the Czech Republic, and India [10, 11, 14–16]. Furthermore, in the Czech Republic, using F-57 or IS*900* real-time PCR, MAP was detected in 49% of samples of powdered infant milk, with one study yielding viable MAP [17].

Nevertheless, little was published regarding MAP in dairy products other than liquid of powdered milk and some cheese. In general, fermented milk products provide significant barriers to pathogen's growth, starting with heat treatment during pasteurization, followed by the addition of a starter culture and the acidity environment during fermentation as well as refrigerated storage. van Brandt et al. inoculated MAP into ultrahigh temperature (UHT) milk and observed that initial MAP counts, during yogurt fermentation followed by storage at 6°C, remained unchanged for over 6 weeks, regardless of the starter culture that was used or the fat content of milk [18].

It is unknown whether the treatment to which milk is subjected to prepare yogurt is sufficient to inactivate viable MAP. Although there are some studies on the behavior of pathogenic bacteria such as *Escherichia coli* (*E. coli*) and *Listeria monocytogenes* during the processing of yogurt, they are affected by different temperatures and low pH. *E. coli *is an important foodborne pathogen and dairy products may contain these bacteria, normally due to postpasteurization contamination. *E. coli *O157:H7, the most studied bacterium of the Enterobacteriaceae family, is able to survive the acidic conditions during yogurt preparation and thus causes bacterial enteric infections to the consumers. Massa et al. [19] found that *E. coli* O157:H7 survived fermentation conditions of low pH levels of 4.5 and at temperatures of 42°C up to 5 h. Bachrouri et al. [20] found that *E. coli* O157:H7 can grow during the preparation of yogurt and survive for 10–21 days at different refrigerated storage temperatures. Several studies have shown that the addition of probiotic cultures in yogurt shortens the survival of *L. monocytogenes* and *E. coli* O157:H7 during the storage period [21–24]. However, not much literature is available about similar studies using *Salmonella* Enteritidis (*S*. Enteritidis).

The aim of this study was to find out the viability of MAP, *E. coli*, and *S*. Enteritidis during the traditional processing and refrigerated storage of yogurt made from milk after experimental inoculation and determine if there are synergistic or antagonistic effects between these bacteria.

## 2. Materials and Methods

### 2.1. Bacteria Strains

Bacterial strains were isolated at the bacteriology laboratory of the National Institute of Agricultural Technology (INTA), Balcarce (Argentina), using MAP strain INTA SB, isolated from a commercial milk, *E. coli *strain INTA 116/C3, obtained from bovine feces, and *S.* Enteritidis strain INTA 86/360, isolated from poultry.

### 2.2. Inoculums Preparation

For the preparation of the inocula, the aforementioned strains were thawed from liquid nitrogen. MAP was cultured onto solid Herrold's egg yolk medium (HEYM) plus 0.0002% (wt/vol) mycobactin J (Allied Monitor Inc., Fayette, MO, USA) and sodium pyruvate (Sigma Chemical Co., St. Louis, MO, USA) for 8 weeks at 37°C. For the preparation of the inoculum, few colonies were suspended in 15 mL of phosphate buffered saline (PBS) (pH = 7), adjusted to turbidity of 0.5 in McFarland scale [25] containing 1.5 × 10^8^ colony forming units (CFU) · mL^−1^. *E. coli* was grown onto McConkey (MC) agar and *S.* Enteritidis was cultured onto Xilosa-Lisina-Desoxicolato agar with the addition of 0.46% of Tergitol-4 (Sigma Chemical Co., St. Louis, MO, USA) (XLDT4). Both MC and XLDT4 agars were incubated overnight at 37°C. Afterwards, one colony of either *E. coli* or *S.* Enteritidis was suspended in PBS pH 7.2 and used to seed brain-heart infusion (BHI) broth (Oxoid, UK), which were further incubated overnight at 37°C. For the preparation of the inocula both BHI tubes were diluted with PBS (pH = 7.2) to a final concentration of 1.5 × 10^8^ colony forming units (CFU) · mL^−1^.

### 2.3. Preparation of Yogurt

Three traditional yogurts were prepared using 360 mL of commercial UHT milk, 125 g of commercial yogurt containing starter culture of *Streptococcus thermophilus *and *Lactobacillus bulgaricus*, and 5 mL of the corresponding bacterial inoculum: *Yogurt 1 (Y1)*: MAP, *Yogurt 2 (Y2)*: *E. coli* + *S.* Enteritidis, and *Yogurt 3 (Y3)*: MAP + *E. coli* + *S.* Enteritidis. The fermentation was carried out in a thermostatic orbital shaker (New Brunswick Scientific, Model E24) at 43°C for 3 h. Afterwards, the yogurts were stored at 4°C for 20 days.

### 2.4. Samples of Yogurt

Samples of 50 mL were taken out before and throughout the preparation time at 20 min, 45 min, 65 min, 90 min, 115 min, 135 min, 155 min, 180 min, and 270 min. Furthermore, samples were also taken during storage at 4.5 h, 24 h, 48 h, 4 days, 10 days, 15 days, and 20 days after inoculation.

### 2.5. Live Bacterial Counts

*MAP*. For decontamination of the samples the method proposed by Tacquet et al. [26] was used with some modifications: 35 mL of each yogurt was centrifuged at 6000 rpm for 20 min in a refrigerated centrifuge (Sigma 16-K, Germany) and the pellet was resuspended in 15 mL of oxalic acid at 5% (wt/vol). The samples were kept at 37°C for 10 min and then centrifuged once more. Afterwards, the pellet was resuspended in 2 mL PBS and finally 160 *μ*L was cultured in HEYM plus vancomycin 0.01% (wt/vol), amphotericin B 5% (wt/vol), nalidixic acid 0.3% (wt/vol), and nystatin 0.01% (wt/vol). The medium was incubated at 37°C for 40 days and was weekly observed for MAP growth. The final CFU was calculated taking into account that each slant was inoculated with 160 *μ*L. The results are expressed as CFU · mL^−1^ of yogurt.

*E. coli and S. Enteritidis*. 5 mL was taken from each yogurt sample and 10 decimal logarithmic dilutions were made in PBS (pH = 7.2). Using the Miles and Misra method [27], an aliquot of 20 *μ*L from each dilution was plated onto triplicate MC and XLDT4 for *E. coli* and *S.* Enteritidis strains, respectively. The agar plates were incubated overnight at 37°C and afterwards colonies were counted in the dilution that permitted the identification of 3–30 separate colonies. The results were expressed as CFU · mL^−1^ of yogurt.

### 2.6. pH Determination

pH was measured using a pH meter (Schott Gerate, CG 820, USA).

### 2.7. Statistical Analysis

The linear regression curves of log counts were analyzed versus time of preparation and storage. The analysis was performed only for *E. coli* and *S*. Enteritidis by comparing the behavior of both bacteria in the yogurt and of each in the two yogurt bacteria which were inoculated. The program Statistical Analysis Software (SAS) was used [28].

## 3. Results

### 3.1. MAP Counts

**Figure 1:**
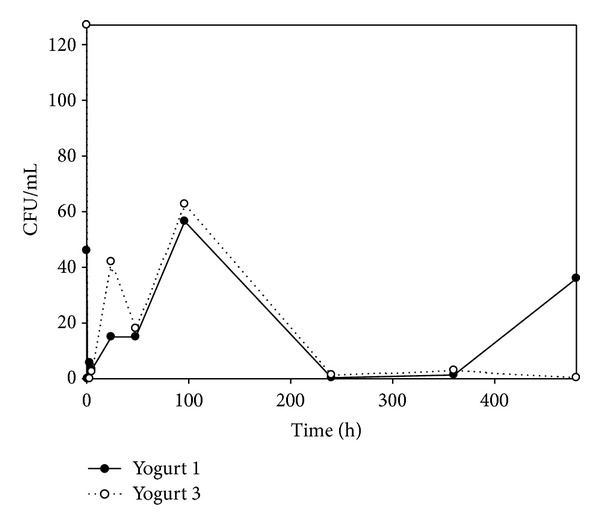
CFU/mL of MAP during preparation and refrigerated storage of yogurts Y1 and Y3.

**Figure 2:**
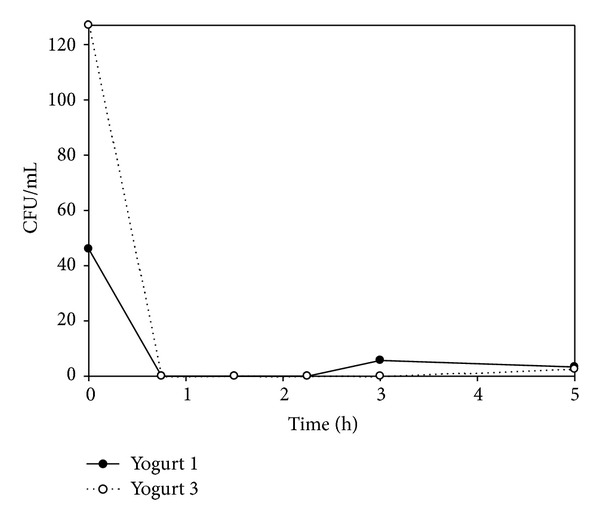
CFU/mL of MAP during the preparation of yogurts Y1 and Y3.

At the beginning of the preparation process, counts of MAP in *Y1* and *Y3* were 46 and 127 CFU · mL^−1^, respectively. During the preparation process (up to 135 min) MAP was not detected in any of the yogurts. After that, MAP counts in *Y1* were 5.6 CFU · mL^−1^ at 180 min, but MAP was not isolated from *Y3*. After 4.5 h and during the storage at 4°C, the CFU · mL^−1^ increased and reached a maximum of 56,5 and 62,7 CFU · mL^−1^ at 4 days in both yogurts, after which the viability began to fall again (Figures 1 and 2).

### 3.2. *E. coli* and *S.* Enteritidis Counts

**Figure 3:**
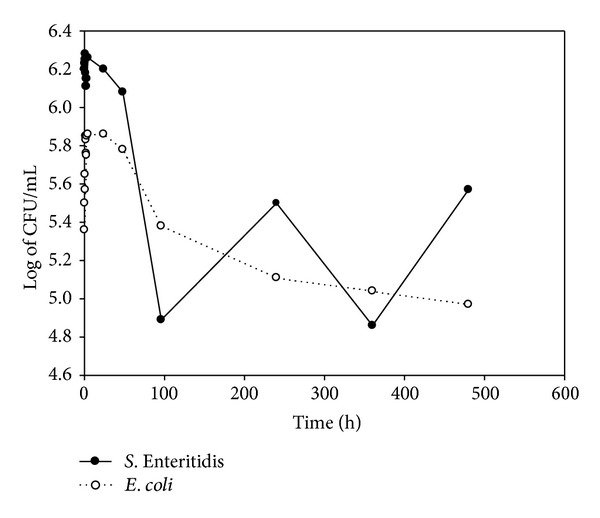
Log of CFU/mL of *E. coli* and *S*. Enteritidis during yogurt preparation and refrigerated storage in Y3.

**Figure 4:**
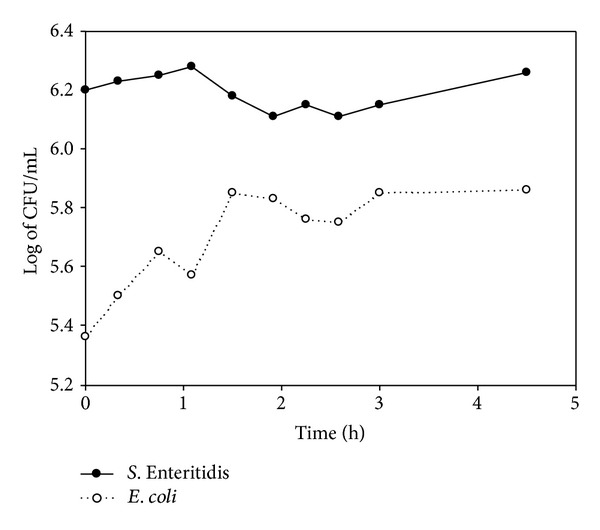
Log of CFU/mL of *E. coli* and *S*. Enteritidis during yogurt preparation in Y3.

**Figure 5:**
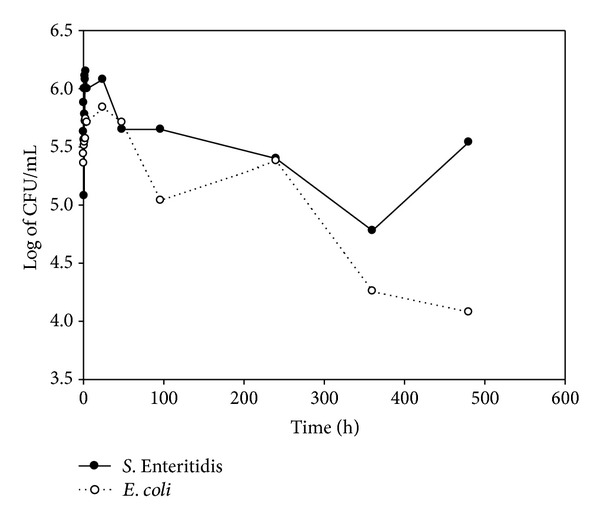
Log of CFU/mL of *E. coli* and *S*. Enteritidis during yogurt preparation and refrigerated storage in Y2.

**Figure 6:**
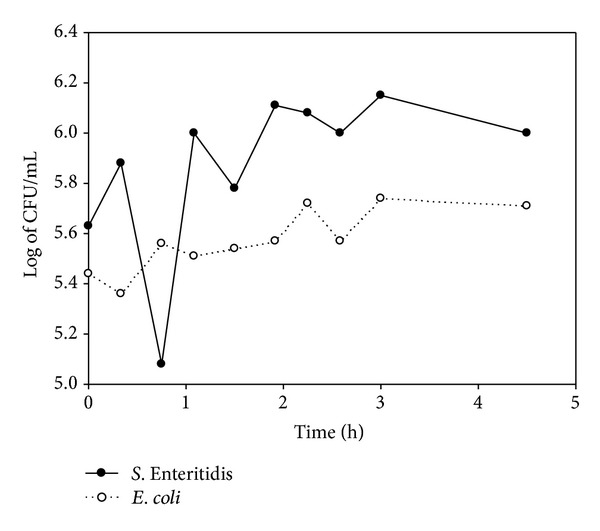
Log of CFU/mL of *E. coli* and *S*. Enteritidis during yogurt preparation in Y2.

The initial counts for *E. coli* and *S. *Enteritidis were 5.3 and 5.5 log of CFU · mL^−1^ and decreased during the first hour of preparation. Both bacteria reached their maximum counts at 24 h (6.4 log of CFU · mL^−1^ for *E. coli* and 5.3 log of CFU · mL^−1^ for *S. *Enteritidis). The viability of both bacteria continued to slightly decline until the end of the trial (5 log for *E. coli* and 4.6 log for *S. *Enteritidis) (Figures 3, 4, 5, and 6).

### 3.3. pH

**Figure 7:**
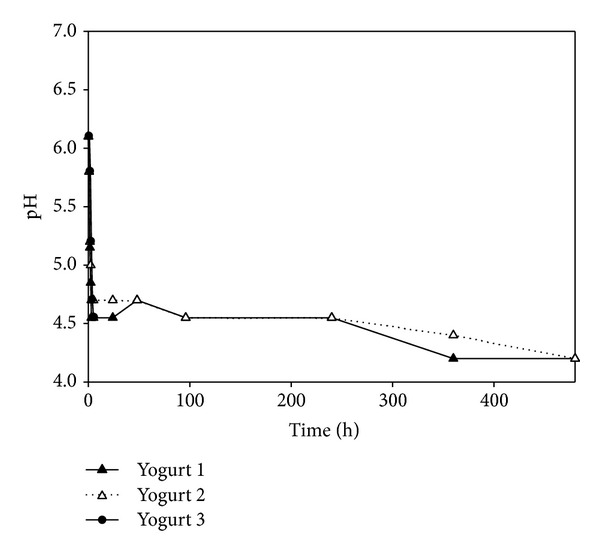
pH evolution during yogurt preparation and refrigerated storage in Y1, Y2, and Y3.

**Figure 8:**
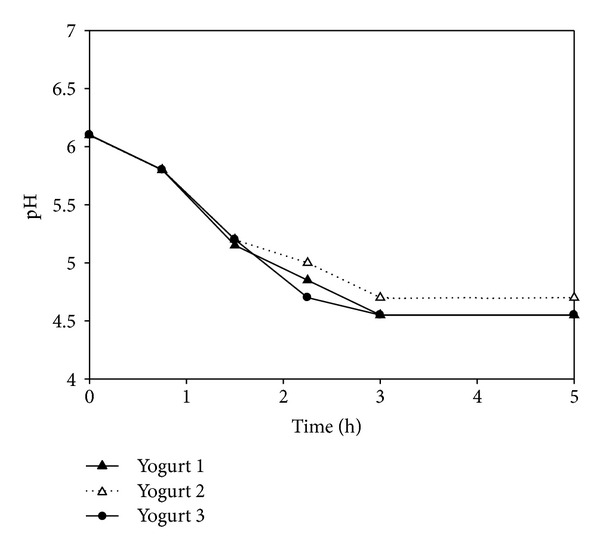
pH evolution during yogurt preparation in Y1, Y2, and Y3.

The initial pH was 6.1 for the three yogurts. During the fermentation process, the pH gradually decreased to 4.5. From 180 min to 4.5 h, values remained in that range. Afterwards, the pH remained stable at 4.5 throughout storage until the end of the trial (Figures 7 and 8).

### 3.4. Statistical Analysis

The regression analysis of the log counts of *E. coli* and *S*. Enteritidis versus the preparation and storage time in *Y3* gave an *R* ^2^ of 0.94 (*P* < 0.05), which shows that the variation in the preparation of storage time is the cause of 94.25% of the variation in the bacterial counts. By comparing the behavior of both bacteria, statistical differences were observed (*P* < 0.05). In *Y2*, the *R* ^2^ was 0.58 indicating that the variation in the preparation and storage time explained 58.27% of the variation in the bacterial counts. In this case, the behaviors of both bacteria were not statistically different (*P* > 0.05). On the other hand, the behavior analysis of *E. coli* in *Y3* and *Y2* showed no significant differences (*P* > 0.05). Instead, *S*. Enteritidis showed a different behavior in both yogurts (*P* < 0.05), which occurs in *Y3* inoculated with MAP.

## 4. Discussion

### 4.1. Growth of MAP

As milk is a nutrient medium suitable for growth of microorganisms, raw milk can be an important source of pathogens for humans. The ability of MAP to survive certain food processing methods and to persist unfavorable conditions, combined with its possible involvement in Crohn's disease, has raised concern with respect to human exposure to this pathogen. For the commercial production of yogurt and other fermented milk products, raw milk is usually first pasteurized. However, the complete elimination of MAP by pasteurization is still under debate. If indeed MAP is present in pasteurized milk, it might be transferred to humans upon consumption of by-products. Therefore, it is interesting to gain insights into the behavior of MAP in such products as the acidic nature of yogurt (pH about 4–4.5) generally contributes to the inactivation of bacterial pathogens [18].

MAP was isolated until the end of the present study (20 days of storage), showing its capacity to withstand the acidic conditions generated during yogurt preparation and the low temperatures of storage. In all samples, MAP counts were low due to the aggressive step of decontamination. van Brandt et al. [18] found that initial MAP counts remain unchanged over the 6 weeks of refrigerated storage, regardless of starter culture used and the fat content of milk, but did not evaluate its isolation during preparation. Sung and Collins [29] found that at low pH levels* in vitro*, MAP expressed two proteins related at the acid tolerance. However, they could not predict MAP survival curve in foods where other factors might affect its viability. In the present work, MAP was not isolated during the preparation process, which suggests that the sudden decrease in pH might have caused damage to the bacteria and therefore it was impossible to culture it on artificial culture media. Furthermore, MAP was recuperated at 4.5 h after inoculation suggesting that initially it might have been in a viable noncultivable state and after an adaptation period.

In a previous work, during the preparation and storage of cheese made with caprine and bovine milk, MAP was isolated only when nonpasteurized milk was used up to 60 days of maturation. High values of pH were observed between 30 and 45 days of storage, coinciding with the further MAP isolation [30]. It has also been observed that pH, temperature, and salt concentrations can affect MAP viability [31–33].

### 4.2. Growth of *E. coli* and *S*. Enteritidis

#### 4.2.1. Preparation

To our knowledge, there are no referential studies on the survival of *S*. Enteritidis during the preparation of dairy products like yogurt. However, some studies have focused on *E. coli*, especially the pathogenic serotype O157:H7, which causes hemolytic uremic syndrome. Bachrouri et al. [20] observed that *E. coli* O157:H7 counts increased during fermentation, whereas Dineen et al. [22] found that this bacterium was not recovered during yogurt fermentation. Therefore, in this study, high inoculation levels were chosen in order to assure recuperation of both *E. coli *and *S.* Enteritidis at the entire process of production and conservation of the yogurts. Hence, it was demonstrated that *E. coli* counts had decreased slowly, while *S*. Enteritidis maintained stable. These differences may be explained by the fact that in these abovementioned works the fermentation conditions were different: higher fermentation temperature (46–7°C), longer fermentation time of 24 h, and lower final pH (4.1). Similarly, Soudah et al. [34] observed that *E. coli* O157:H7 did not survive fermentation in yogurt containing *Lactobacillus delbrueckii* subsp. *bulgaricus* and *Streptococcus thermophilus *at 47°C. However, other researchers found that *E. coli* O157:H7 survived the fermentation of yogurt at 42°C [19, 23]. Furthermore, the exposure to acid environments for short times increased the tolerance acid of *E. coli*, thus permitting survival for longer periods [35]. These results are in agreement with those of the present work, where, despite suffering a slight decline, *E. coli* was recovered during the fermentation process at 43°C and a final pH of 4.7. These results suggest that the conditions of fermentation are important factors that may affect the survival of *E. coli*.

#### 4.2.2. Storage

Bachrouri et al. [20] observed that viability of *E. coli* began to decline until 7 days of storage, when no viable bacteria were detected. Barrantes et al. [21] found that no viable *E. coli* O157:H7 was detected in yogurts made with and without probiotics, at 16 and 28 days of storage, respectively. Govaris et al. [23] did not detect viable *E. coli* O157:H7 between 5 and 8 days of storage at 4°C or between 4 and 6 days of storage at 12°C and the pH of the yogurts was stable. However, in the present work, using a similar inoculation dose in the yogurts, *E. coli* and *S.* Enteritidis were isolated at 20 days of cold storage. This reveals the capacity of these bacteria to tolerate acidity conditions when they are present in raw material. On the other hand, lactic bacteria produce compounds such as organic acids, hydrogen peroxide, diacetyl, and bacteriocins, which inhibit the growth of contaminant microflora and pathogens such as* Staphylococcus aureus*, *Clostridium botulinum*, and *L. monocytogenes *[24]. In the present study, it is suggested that the presence of MAP in *Y3* contributed to the reduced survival of *S.* Enteritidis in comparison with *Y2*. However, the mechanism by which these changes occur was not studied. There is also a lack of information about the competitive inhibition of MAP and other bacteria for nutrients in the yogurt during preparation. Nevertheless, if such competition does occur, MAP would be in disadvantage as it is the cultivable mycobacterium with the slowest growth, with a slow generation time of over 20 h, under optimal growth conditions, whereas *S.* Enteritidis has a generation time of 20 minutes [36].

Further studies should be performed to assess the competitive inhibition mechanisms of these pathogens *in vitro *as well as their resistance to different temperatures and pH levels in order to improve optimal conditions for the processing of dairy products and to ensure their elimination.

## 5. Conclusions

The present study presents evidence that pathogen bacteria like MAP,* E. coli*, and *S.* Enteritidis can survive yogurt fermentation conditions and low pH levels followed by refrigerated storage for at least 20 days. Thus these pathogens are present in raw materials and they might reach consumers. The importance of implementing good manufacturing practices during the production and storage of yogurt is highlighted. Therefore, similar studies should be carried out with different concentrations of bacteria, in an attempt to simulate natural contamination.
